# Epidemiological and Genomic Characterization of *Campylobacter jejuni* Isolates from a Foodborne Outbreak at Hangzhou, China

**DOI:** 10.3390/ijms21083001

**Published:** 2020-04-24

**Authors:** Hua Yu, Mohammed Elbediwi, Xiaohong Zhou, Huiqun Shuai, Xiuqin Lou, Haoqiu Wang, Yan Li, Min Yue

**Affiliations:** 1 Hangzhou Center for Disease Control and Prevention, Hangzhou 310021, China; indexofire@gmail.com (H.Y.); jolly3360@tom.com (X.L.); wanghaoqiu@126.com (H.W.); 2 Institute of Preventive Veterinary Sciences & Department of Veterinary Medicine, Zhejiang University College of Animal Sciences, Hangzhou 310058, China; m.elbediwi@zju.edu.cn (M.E.); yanli3@zju.edu.cn (Y.L.); 3 Animal Health Research Institute, Agriculture Research Centre, Cairo 11865, Egypt; 4 Xiacheng Center for Disease Control and Prevention, Hangzhou 310003, China; zxh1219288551@hotmail.com (X.Z.); qbshq@163.com (H.S.); 5 Zhejiang Provincial Key Laboratory of Preventive Veterinary Medicine, Hangzhou 310058, China

**Keywords:** *Campylobacter jejuni*, foodborne outbreak, genomic investigation, pulse field gel electrophoresis, ST2988

## Abstract

Background: Foodborne outbreaks caused by *Campylobacter jejuni* have become a significant public health problem worldwide. Applying genomic sequencing as a routine part of foodborne outbreak investigation remains in its infancy in China. We applied both traditional PFGE profiling and genomic investigation to understand the cause of a foodborne outbreak in Hangzhou in December 2018. Method: A total of 43 fecal samples, including 27 sick patients and 16 canteen employees from a high school in Hangzhou city in Zhejiang province, were recruited. Routine real-time fluorescent PCR assays were used for scanning the potential infectious agents, including viral pathogens (norovirus, rotavirus, adenovirus, and astrovirus), and bacterial pathogens (*Salmonella*, *Shigella*, *Campylobacter jejuni*, *Vibrio parahaemolyticus* and *Vibrio cholerae*). Bacterial selection medium was used to isolate and identify the positive bacteria identified by molecular test. Pulsed field gel electrophoresis (PFGE), and next generation sequencing (NGS) were applied to fifteen recovered *C. jejuni* isolates to further understand the case linkage of this particular outbreak. Additionally, we retrieved reference genomes from the NCBI database and performed a comparative genomics analysis with the examined genomes produced in this study. Results: The analyzed samples were found to be negative for the queried viruses. Additionally, *Salmonella, Shigella, Vibrio parahaemolyticus and Vibrio cholera* were not detected. Fifteen *C. jejuni* strains were identified by the real-time PCR assay and bacterial selection medium. These *C. jejuni* strains were classified into two genetic profiles defined by the PFGE. Out of fifteen *C. jejuni* strains, fourteen have a unified consistent genotype belonging to ST2988, and the other strain belongs to ST8149, with a 66.7% similarity in comparison with the rest of the strains. Moreover, all fifteen strains harbored *bla_OXA-61_* and *tet(O)*, in addition to a chromosomal mutation in *gyrA* (T86I). The examined fourteen strains of ST2988 from CC354 clone group have very minimal genetic difference (3~66 SNPs), demonstrated by the phylogenomic investigation. Conclusion: Both genomic investigation and PFGE profiling confirmed that *C. jejuni* ST2988, a new derivative from CC354, was responsible for the foodborne outbreak Illustrated in this study.

## 1. Introduction

*Campylobacter jejuni* is a common foodborne pathogenic bacterium which causes gastroenteritis, and more severely, a neural damage disease in humans called Guillain-Barre syndrome [1]. Raw milk, water, and contaminated meat, particularly chicken are believed to be the main sources of *C. jejuni* human infections [2,3].

*C. jejuni* is considered to be the leading cause of human gastroenteritis [4] and ranked as the second important cause for foodborne diseases in the U.S., with more than 1.5 million illness annually according to the Centers for Disease Control and Prevention (CDC), it has also been reported as one of the most commonly described pathogens in humans in the European Union foodborne disease surveillance network since 2005 [5,6]. Recently, there has been a surge in the global incidence of *Campylobacter* infections, and ongoing spread of human cases in North America, Europe, and Australia [7]. Though foodborne disease caused by *C. jejuni* has become an important public health concern, there is limited knowledge about its role in foodborne disease outbreaks in China. This knowledge gap could be due to *Campylobacter* infections not being subjected to obligatory reports and its surveillance being on a voluntary basis by local and regional laboratories.

The pulsed field gel electrophoresis (PFGE) has been widely used in outbreak investigations for tracking sources of infection and effectively controlling epidemics due to its good reproducibility, high resolution and stable results, and the ease of standardization [8]. Nowadays, next generation sequencing (NGS) technology is becoming popular, considering advantages of labor- and time-saving, high-throughput capacities, highly precise and abundance of genetic information available for extensive studies. As the sequencing cost continues to decrease, genomic epidemiology combined with NGS has been increasingly and widely applied to outbreak investigations [9,10]. The PFGE technology and other genotyping approaches, including multi-locus sequence typing (MLST), shows that *Campylobacter* is not a genetically monomorphic organism, but includes highly diverse assemblies with an array of different phenotypes [9,10,11]. Considering this complexity, there are sufficient genetic materials, which could be used to link a particular genotype with a certain animal host [2,12]. Nevertheless, few *C. jejuni* Chinese clinical isolates with genome sequence are available in the public genomic database. The aim of this study was to describe both the epidemiological investigation and genomic characterization of *C. jejuni* that was responsible for the outbreak in a high school in Hangzhou in December 2018 using PFGE and NGS technologies.

## 2. Results

### 2.1. Causative Pathogen Scanning

All forty-three samples were found negative for norovirus, rotavirus, adenovirus, sapovirus and astrovirus. Additionally, *Salmonella, Shigella*, *Vibrio parahaemolyticus,* and *Vibrio cholerae* were also not detected in all the examined patients. Fifteen strains of *C. jejuni*, from the fifteen sick students (Table 1), were identified by real-time fluorescent PCR and confirmed by the traditional microbiological approaches.

### 2.2. PFGE Profiling Studies

**Figure 1:**
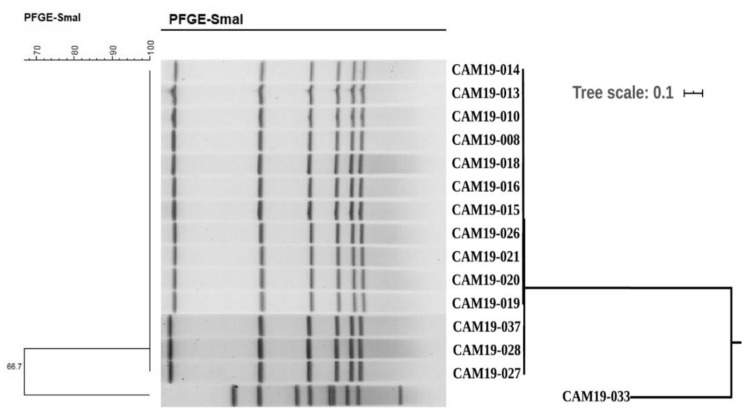
PFGE dendrogram and phylogenomic SNPs tree of the fifteen strains of *C. jejuni*. The PFGE-*Sma*I enzyme digestion patterns are shown on the left and the middle. The phylogenomic SNPs tree of the fifteen strains of *C. jejuni* is shown on the right with a scale bar at 0.1.

The fifteen *C. jejuni* strains were classified into two types, PA1 (fourteen strains) with an overall 99.7% similarity, and PA2 (one strain). The similarity between PA1 and PA2 was 66.7% (Figure 1, Table 1).

### 2.3. Genomic Sequencing

After conducting the whole genome sequencing and genomic assembly of the *C. jejuni* strains, the number of contigs was calculated to be between 12 and 48 contigs. Genome sequencing, assembly results and accession number are summarized in Table 1. The average genome size of draft assemblies was 1,650,982. Furthermore, the average N50 was 255,161 with 30.33% as average of GC%.

**Figure 2:**
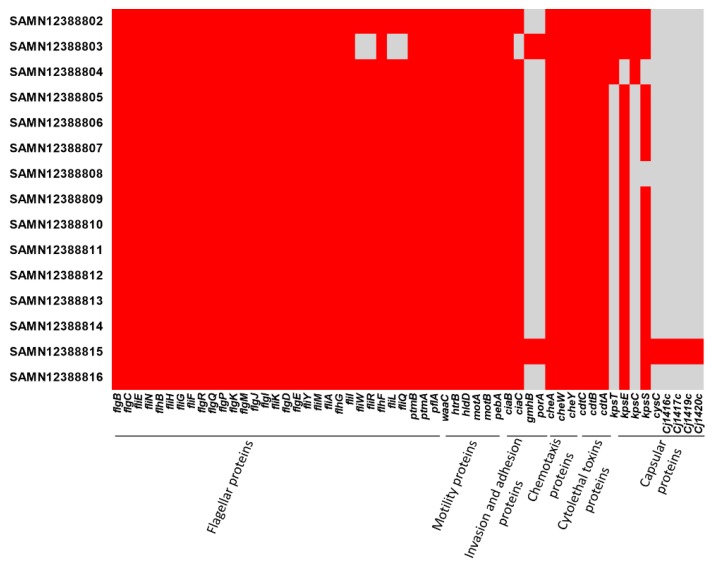
Virulence factors detected in the examined *C. jejuni* isolates in this study. Red color refers to presence of the protein and grey color refers to absence of the protein.

The assembly results were scanned and identified with their MLST profiles. Fourteen strains of *C. jejuni* belonged to ST2988 and only one strain belonged to the ST8149 type. Further analysis showed that all the strains harbored *bla_OXA-61_* which encodes resistance to β-lactamases, and *tet(O)* which confers resistance to tetracyclines. Additionally, a chromosomal mutation in *gyrA* (T86I), which might be responsible for the resistance to fluoroquinolones, was detected in all fifteen strains. No plasmid replicons were detected in any isolate. Furthermore, Figure 2 shows that all isolates harbored flagellar, motility, chemotaxis and cytolethal toxin proteins. SAMN12388815 isolate harbored *gmhP*, *porA* proteins which play a vital role in bacterial virulence by enhancing the adhesion and invasion properties. Cysc, Cj1416c, Cj1417c, Cj1419c, Cj1420c proteins, which are involved in capsule polysaccharide biosynthesis, were only detected in one strain (SAMN12388815). We also identified that both *kpsT* and *kpsC* proteins, which were involved in capsule polysaccharide biosynthesis, were only in three strains (SAMN12388802, SAMN12388803, SAMN12388804, in Figure 2).

### 2.4. Genome Comparison and Phylogenomic Analysis 

The phylogenomic tree shows that all fourteen case-patient isolates from this particular outbreak, which belonged to ST2988, are closely related and clustered together in a single clade. The other individual strain belonged to ST8149 (Figure 1). Importantly, the genomes of these fourteen ST2988 isolates were differed in (< 70) core SNPs, and showed (> 99%) a high similarity (Table S1).

**Figure 3:**
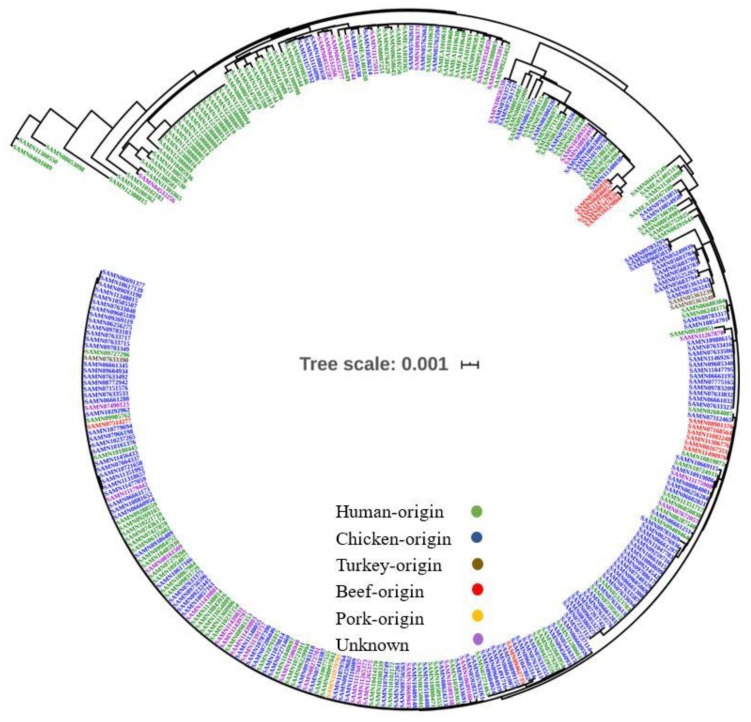
Phylogenomic tree of CC354 associated genomes in *C. jejuni*. Green color refers to human-origin isolates, blue color refers to chicken-origin isolates, orange color refers to pork-origin isolates, red color refers to beef-origin isolates, brown color refers to turkey-origin isolates and violet color refers to the unknown source.

**Figure 4:**
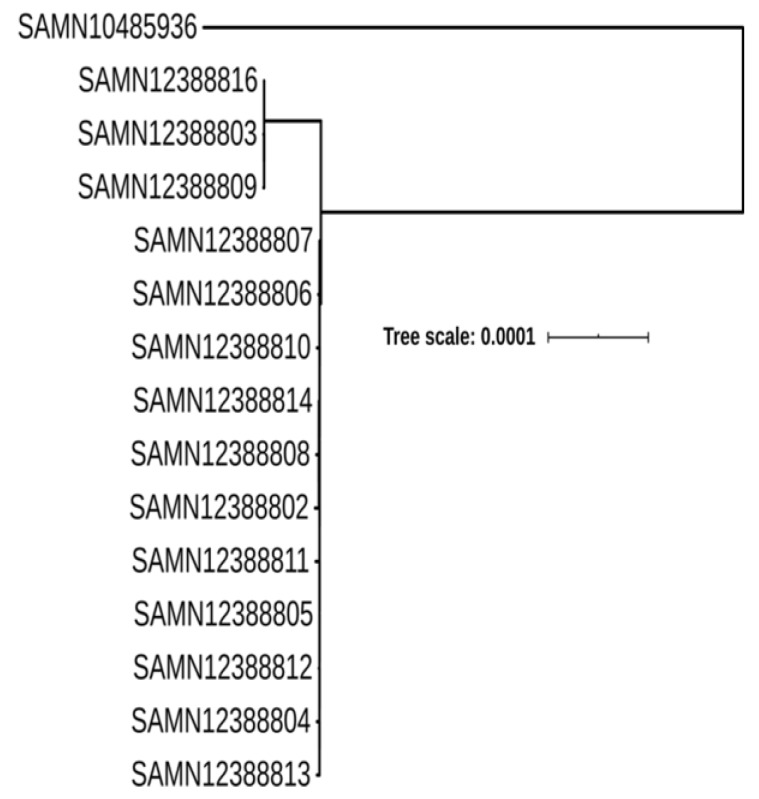
Phylogenomic relationship between 14 isolates linked with the outbreak in this study, and the only available genome ST2988 (SAMN10485936) in the NCBI database. The scale bar is at 0.0001.

The ST2988 belongs to CC354 that includes 199 identified sequence types in (http://pubmlst.org/campylobacter/). Genomic data of all CC354 strains in NCBI database were extracted and 303 genomes were obtained (Table S2), including 27 sequence types. With ST354 strain RM1221 (GCA_000011865.1) as the reference genome, the SNP locus and phylogenetic tree between 302 strains in the public database and 14 ST2988 isolates from this outbreak were obtained (Figure 3). ST354 is the most predominant sequence type in CC354 (Figure S1). Most CC345 isolates were isolated from humans and food samples, and few isolates were retrieved from unknown sources (Figure 3, and Figure S1). Isolates from chicken-origin were identified to have the highest prevalence among the food isolates (Figure 3, and Table S2). The CC354 strains in the public databases are mainly from the US and the UK (Figure S1), while strains from other countries are scattered. A small difference in distance between phylogenetic branches of CC345 isolates was identified in Figure 3 with a scale bar at 0.001, indicating a very close genetic relationship within the sequence type. We also observed a close relationship with a scale bar at 0.001 among these 14 strains linked with the outbreak in this study, which were also linked with the only available genome (SAMN10485936) in the NCBI database (Figure 4).

## 3. Discussion

Recently, the rate of *Campylobacter* infections has rapidly increased due to the expansion of the consumption of raw or undercooked chicken, especially in China [13]. In December 2018, a serious case of foodborne disease was reported in a high school, where eighty-four students in twelve classes from grade one to six had diarrhea, vomiting, fever and other foodborne disease-associated symptoms, in Hangzhou. To identify the causative agent of this outbreak, 43 fecal samples were collected from patient students and canteen workers. Nucleic acid of suspected viral or bacterial samples were extracted for laboratory investigation. None of these samples were positive for the suspected viruses. Fifteen strains of *C. jejuni* were detected and isolated from the samples of fifteen sick students. To the best of our knowledge, this is the second foodborne outbreak of *C. jejuni* described in China to date. The previous outbreak led to 36 cases of *Campylobacter* infections that occurred in a high school in Beijing after a trip to another province in Southern China [14].

In order to provide more reliable evidence for the outbreak origin, we conducted PFGE profiling and genomic analysis for these fifteen strains of *C. jejuni*, which is essential for evaluating the clinical isolates from the outbreak and related cases [15]. The results showed that these fourteen strains belonged to the same pattern (PA-1), while the one other strain which had a similarity of 66.7%, belonged to the other pattern (PA-2). By using genomic data for MLST or genotype scanning, it was found that 14 strains were of ST2988 type and one of ST8149 type, which was consistent with PFGE results. These results suggested that the unique ST2988 *C. jejuni* isolate was responsible for this foodborne outbreak. Scrutiny of the PFGE pattern (PA-1) exhibited an inherent similarity, with some changes in three isolates (CAM19-027, CAM19-028, CAM19-037) belonging to the same MLST (Figure 1), which hints towards a recent evolutionary deviation from a common ancestor. Although these isolates had a slightly deviant PFGE pattern, it was not considered significant enough to exclude them from this outbreak, as the variations in the PFGE patterns can result from a single-nucleotide polymorphism in a restriction site [16]. Thus, a clonal relationship may be found even between strains with dissimilar PFGE profiles. Furthermore, a PFGE profile can change after only a single passage through the host by genomic rearrangement [17]. Such changes may occur at relatively high frequency by the discriminatory power of PFGE, compared with MLST, and do not exclude our conclusion regarding the source of infection [18], considering that genotyping results are always in the context of other results from the outbreak investigation.

There are limited epidemiological studies reported on *C. jejuni* ST2988 in China. This particular sequence type has only been reported in three (0.25%) strains from poultry in Jiangsu province, a province close to Zhejiang province in 2014 [19]. Interestingly, there are only two strains belonging to ST2988 from the unknown sources: One strain was in the UK, and the other strain was from the US, as described in the *Campylobacter* PubMLST database (http://pubmlst.org/campylobacter/), an additional strain GCA_004825105.1 (PNUSAC006969, Biosample: SAMN10485936, in October 2018 from a patient aged 40–49) was also described in the NCBI database. As shown in Figure 4, we found a close relation with the 14 strains isolated in this study and only one available genome (SAMN10485936) in the NCBI database with a scale bar at 0.0001.

This ST2988 belonged to CC354, which included 2707 isolates submitted to PubMLST, with a total of 199 different sequence types (http://pubmlst.org/campylobacter/), although only three isolates of *C. jejuni* ST2988 were found in the public database. The CC354 strains in the public databases are mainly from the US and the UK (Figure 3), while the submitted isolates in other countries are scattered. However, CC354 is frequently associated with human clinical infections (47.9%) and poultry (30.7%) (http://pubmlst.org/campylobacter/), it has also been indicated from wild birds in Spain [20], ducks in South Korea [21] and from cattle and pig carcasses in Poland [22]. Large surveillance data on *C. jejuni* isolates from humans as well as various other animals could provide additional knowledge of disease ecology and host reservoirs, which might aid in source attribution for this particular outbreak.

Genome MLST types of a total of 303 strains of high-quality CC354 were retrieved from the NCBI assembly public database and were used to conduct the comparative genomics analysis. We found that there is very limited genetic difference in the distance between the branches of the evolutionary tree of CC345 isolate genomes, indicating an obvious consistency with the sequence type results. This information demonstrates that MLST genotyping based on the housekeeping gene is correlated with their genomic phylogeny.

The mechanisms by which *Campylobacter* species cause diarrhea, and knowledge for the following sequelae are lacking [23]. The genes associated with bacterial motility, invasion and adhesion to epithelial cells, which are critical in the development of *Campylobacter* infection [24,25], were detected in all isolates. These findings confirmed the evidence that flagellar and adhesion genes are highly conserved among *C. jejuni*, as previously reported [23,26]. Furthermore, virulence marker determinants included *cdtA, cdtB, and cdtC* cytotoxin genes, which play an important role in diarrhea by interfering with the division and differentiation of the intestinal crypt cells, were also identified in all examined isolates. As it has been shown in previous investigations, all three subunits are required for full toxin activity [23].

*Campylobacter* is a major foodborne pathogen, and its resistance to clinically vital antibiotics is posing a significant health concern [4,27,28]. Particularly, rising fluoroquinolones and tetracyclines resistance in *Campylobacter* have been reported in many countries [4]. Fluoroquinolones are considered to be the rational drug of choice in treating human campylobacteriosis [12,29], but in certain cases, tetracyclines are used to treat systemic infection caused by *Campylobacter* [12,27]. Genomic analysis in this study indicated that all the tested isolates harbored *tet(O)* which confer resistance to tetracyclines, and a chromosomal mutation in *gyrA* (T86I) which confer resistant to fluoroquinolones. Resistance to these two antibiotics were also the most frequently reported in *Campylobacter* infections in China [30,31,32]. More than 90% of the *Campylobacter spp*. isolates have been reported to be resistant to quinolones and tetracycline in Shanghai, also in eastern China [33]. Furthermore, *C. jejuni* strains obtained from retail chicken meat samples have been described with high resistance to ciprofloxacin and tetracycline in central China [34]. As antimicrobial resistance tenders a significance alarm [35], substantial concern should be given to the antimicrobial resistance in *C. jejuni*. A long-term monitoring system is needed for improved control of infections, epidemics and antimicrobial resistance to crucial antimicrobials for bacterial agents, including *C. jejuni*.

## 4. Material and Methods

### 4.1. Epidemiological Investigation

In December 2018, a series of patients reported foodborne diseases in a high school in Hangzhou, the capital city of Zhejiang province in eastern China. Eighty-four students, in twelve classes from grade one to six, complained of symptoms of food poisoning. No meals were served at the school other than school lunches, which could be the potential source of this foodborne outbreak.

We defined a probable case as a patient with diarrhea, vomiting or other symptoms (abdominal pain, fever and so on) and a confirmed case as a patient with any symptoms and a confirmed laboratory diagnosis of *C. jejuni*.

### 4.2. Samples Collection

Local CDC microbiologists collected 43 fecal samples based on the Chinese local regulations, of which 27 were from sick students and 16 from canteen employees, as probable cases for microbiological investigation. Canteen food samples were disposed of by the head of school due to the concerns of further contamination and disease dissemination, so no foods were available in the current investigation.

### 4.3. Pathogen Detection

Real-time fluorescent PCR was used to detect norovirus, rotavirus, adenovirus, sapovirus and astrovirus according to a protocol reported earlier [36]. WS271-2007 diagnostic criteria for infectious diarrhea protocol [37,38] was used for the detection of *Salmonella, C. jejuni* and *Vibrio parahaemolyticus*. WS287-2008 [39] and WS289-2008 [40] protocols were used for detection of *Shigella* and *Vibrio cholerae*, respectively. Briefly, fecal samples were added to an Eppendorf tube with sterile saline to prepare a stool suspension. Total genomic DNA, including bacterial and viral agents, was extracted and purified from the stool suspension using QIAamp DNA mini Kit (Qiagen, Hilden, Germany, No: 51304), according to the manufacturer’s recommended protocols. Real-time fluorescent PCR was performed at 42 °C for 1 h and 95 °C for 15 min, followed by 40 cycles of 94 °C for 60 s, 58 °C for 80 s, and 72 °C for 60 s, with a final extension at 72 °C for 7 min.

### 4.4. Isolation and Identification of Campylobacter spp.

The positive *Campylobacter* samples detected by the real-time fluorescent PCR were pre-enriched with Preston selective broth supplemented with 5% sterile, lysed sheep blood, *Campylobacter* growth supplement and selective supplement (Oxoid Ltd., Basingstoke, UK). Samples were incubated at 42 ℃ under microaerobic conditions (5% O2, 10% CO2, and 85% N2) for 12–24 h. Two hundred microliter drops of the pre-enrichment were applied to the 0.45-μm pore-size filter and left on the surface of a Columbia blood agar plate. These plates were further incubated at 37 ℃ under microaerobic conditions [41].

### 4.5. Pulsed Field Gel Electrophoresis (PFGE) Testing

PFGE molecular typing was performed according to the PFGE protocol for *C. jejuni* [42,43]. Briefly, restriction digestion was conducted by using 40 U *Sma*I (Takara, Dalian, China), and run on a CHEF Mapper PFGE system (Bio-Rad Laboratories, Hercules, Canada) for SeaKem gold agarose (Lonza, Rockland, MD, USA) in 0.5×Tris-borate-EDTA. Bionumerics v6.6 software was used for the clustering analysis. Similarity greater than 95% was considered as the same genetic group. The similarity between chromosomal fingerprints was scored using the Dice coefficient. The unweighted pair group method, with arithmetic means (UPGMA) at the cut-off of 1.5% tolerance and 1.00% optimization, was used to obtain the dendrogram in the PFGE profile.

### 4.6. Genomic Sequencing and Bioinformatic Analysis

The Genomic DNA library was constructed using Nextera XT DNA library construction kit (Illumina, USA, No: FC-131-1024); followed by genomic sequencing using Miseq Reagent Kit v2 300cycle kit (Illumina, USA, No: MS-102-2002). High-throughput genome sequencing was accomplished by the Illumina Miseq sequencing platform, as previously described [44,45,46]. The quality of sequencing and trimming was checked with FastQC toolkit, while low-quality sequences and joint sequences were removed with trimmomatic [47]. The genome assembly was performed with SPAdes 4.0.1 for genomic scaffolds [48], using the “careful correction” option in order to reduce the number of mismatches in the final assembly with automatically choosen *k*-mer values by SPAdes. QUAST [49] was used to evaluate the assembled genomes through basic statistics generation, including the total number of contigs, contig length, and N50. Prokka 1.14 [50], with the “default” settings was used to annotate the assembled genomes. Multilocus sequence typing (MLST) software (http://www.github.com/tseemann/mlst) was applied for the sequence type of the isolates for the in-house database. Detection of resistance genes, plasmids replicons and virulence genes were conducted using ABRicate software (http://www.github.com/tseemann/abricate). All the sequence types from a clonal complex (CC) detected by using the genome sequence were retrieved from the NCBI assembly database. Considering RM1221 strain [51] as a reference genome, we used two different protocols to conduct the multiple sequence alignment of the genomes in order to build the phylogenomic tree, and both of them delivered the identical results. The first approach was performed using Snippy to search for single nucleotide polymorphism (SNP) locus [52]. The second approach was conducted by Gubbins to produce the consensus sequence, and Mafft was used to make the multiple sequence alignment for the whole genome sequences [52]. The phylogenomic tree was built and projected with RAxML [53] and ITOL [54], respectively.

### 4.7. Ethical Approval

All procedures performed in studies involving human participants were officially approved by the Xiacheng CDC at Hangzhou (No. 2019-05, 20190716), which was in accordance with the ethical standards of the institutional research committee and with the 1964 Helsinki declaration and its later amendments or comparable ethical standards.

## 5. Conclusions

This analysis sheds light on the possible menace of *C. jejuni* infections. PFGE and NGS technologies provided reliable evidence for the identification of the pathogens for this outbreak, caused by *C. jejuni* ST2988. These results suggest that enhanced concerns should be given to the circulation of this rarely reported sequence type. It is expected that the advanced NGS technologies will be promising in pathogen detection and foodborne disease tracking.

To our knowledge, this is the second *C. jejuni* outbreak described in China to date. Unfortunately, in this event, food samples were not included in the investigation. In the future, the collection and testing of food samples should be emphasized for a more comprehensive investigation. These data also endorse that authorities need to implement systematic surveillance and compulsory notification for *Campylobacter* infections from humans as well as different animals, which is essential for the identification and tracking of the source of infection and the rationalization of effective control measures to ensure public health and safety.

## Figures and Tables

**Table 1:** Overview of the genome sequences of *Campylobacter jejuni* isolates.

No.	Strains	Samples	ST*	PFGE	Genome Size	GC%	Contigs	N50	Accession
1	CAM19-008	Patient8	2988	PA1	1,655,630	30.39	19	242,158	SAMN12388802
2	CAM19-010	Patient10	2988	PA1	1,652,624	30.39	13	332,935	SAMN12388803
3	CAM19-013	Patient13	2988	PA1	1,645,702	30.36	20	188,661	SAMN12388804
4	CAM19-014	Patient14	2988	PA1	1,649,319	30.35	48	69,212	SAMN12388805
5	CAM19-015	Patient15	2988	PA1	1,645,746	30.36	18	242,282	SAMN12388806
6	CAM19-016	Patient16	2988	PA1	1,644,870	30.35	19	255,151	SAMN12388807
7	CAM19-018	Patient18	2988	PA1	1,648,275	30.34	18	255,143	SAMN12388808
8	CAM19-019	Patient19	2988	PA1	1,647,897	30.34	20	255,112	SAMN12388809
9	CAM19-020	Patient20	2988	PA1	1,654,258	30.39	22	183,324	SAMN12388810
10	CAM19-021	Patient21	2988	PA1	1,645,811	30.36	18	179,689	SAMN12388811
11	CAM19-026	Patient26	2988	PA1	1,645,644	30.35	22	207,949	SAMN12388812
12	CAM19-027	Patient27	2988	PA1	1,650,856	30.34	42	112,156	SAMN12388813
13	CAM19-028	Patient28	2988	PA1	1,651,306	30.40	12	333,007	SAMN12388814
14	CAM19-033	Patient33	8149	PA2	1,678,861	30.49	21	120,767	SAMN12388815
15	CAM19-037	Patient37	2988	PA1	1,647,931	30.34	17	255,111	SAMN12388816

## Supplementary Materials

Supplementary materials can be found at https://www.mdpi.com/1422-0067/21/8/3001/s1.

## References

[1] Zhang M., Li Q., He L., Meng F., Gu Y., Zheng M., Gong Y., Wang P., Ruan F., Zhou L. (2010). Association Study Between an Outbreak of Guillain-Barre Syndrome in Jilin, China, and Preceding *Campylobacter jejuni* Infection. Foodborne Pathog. Dis..

[2] Silva J., Leite D., Fernandes M., Mena C., Gibbs P.A., Teixeira P. (2011). *Campylobacter* spp. as a Foodborne Pathogen: A Review. Front. Microbiol..

[3] Paudyal N., Pan H., Liao X., Zhang X., Li X., Fang W., Yue M. (2018). A Meta-Analysis of Major Foodborne Pathogens in Chinese Food Commodities Between 2006 and 2016. Foodborne Pathog. Dis..

[4] Shen Z., Wang Y., Zhang Q., Shen J. (2018). Antimicrobial Resistance in *Campylobacter spp.*. Microbiol. Spectr..

[5] European Food Safety Authority and European Centre for Disease Prevention and Control (EFSA and ECDC) (2018). The European Union summary report on trends and sources of zoonoses, zoonotic agents and foodborne outbreaks in 2017. EFSA J..

[6] Tack D.M., Marder E.P., Griffin P.M., Cieslak P.R., Dunn J., Hurd S., Scallan E., Lathrop S., Muse A., Ryan P. (2019). Preliminary Incidence and Trends of Infections with Pathogens Transmitted Commonly Through Food—Foodborne Diseases Active Surveillance Network, 10 U.S. Sites, 2015–2018. MMWR.

[7] Kaakoush N.O., Castaño-Rodríguez N., Mitchell H.M., Man S.M. (2015). Global Epidemiology of *Campylobacter* Infection. Clin. Microbiol. Rev..

[8] Lahti E., Löfdahl M., Skarin J., Hansson I., Engvall E. (2016). Confirmation of a Campylobacteriosis Outbreak Associated with Chicken Liver Pâté Using PFGE and WGS. Zoonses Public Health.

[9] Duarte A., Seliwiorstow T., Miller W.G., De Zutter L., Uyttendaele M., Dierick K., Botteldoorn N. (2016). Discriminative power of *Campylobacter* phenotypic and genotypic typing methods. J. Microbiol. Methods.

[10] Oakeson K.F., Wagner J.M., Rohrwasser A., Atkinson-Dunn R. (2018). Whole-Genome Sequencing and Bioinformatic Analysis of Isolates from Foodborne Illness Outbreaks of *Campylobacter jejuni* and *Salmonella enterica*. J. Clin. Microbiol..

[11] Carleton H., Gerner-Smidt P. (2016). Whole-Genome Sequencing Is Taking over Foodborne Disease Surveillance: Public health microbiology is undergoing its biggest change in a generation, replacing traditional methods with whole-genome sequencing. Microbe.

[12] Acheson D., Allos B.M. (2001). *Campylobacter jejuni* Infections: Update on Emerging Issues and Trends. Clin. Infect. Dis..

[13] Zeng D., Zhang X., Xue F., Wang Y., Jiang L., Jiang Y. (2016). Phenotypic Characters and Molecular Epidemiology of *Campylobacter jejuni* in East China. J. Food Sci..

[14] Qu M., Zhang M., Zhang X., Jia L., Xu J., Chu Y., Liang Z., Lv B., Liang H., Huang Y. (2019). Molecular and epidemiologyical analysis of a *Campylobacter jejuni* outbreak in China, 2018. J. Infect. Dev. Ctries..

[15] Sheppard S.K., Dallas J.F., Strachan N.J.C., MacRae M., McCarthy N.D., Wilson D.J., Gormley F.J., Falush D., Ogden I.D., Maiden M.C.J. (2009). *Campylobacter* Genotyping to Determine the Source of Human Infection. Clin. Infect. Dis..

[16] Scott A.E., Timms A.R., Connerton P.L., Loc Carrillo C., Adzfa Radzum K., Connerton I.F. (2007). Genome dynamics of *Campylobacter jejuni* in response to bacteriophage predation. PLoS Pathog..

[17] Hänninen M.L., Hakkinen M., Rautelin H. (1999). Stability of related human and chicken *Campylobacter jejuni* genotypes after passage through chick intestine studied by pulsed-field gel electrophoresis. Appl. Environ. Microbiol..

[18] Clark C.G., Price L., Ahmed R., Woodward D.L., Melito P.L., Rodgers F.G., Jamieson F., Ciebin B., Li A., Ellis A. (2003). Characterization of waterborne outbreak-associated *Campylobacter jejuni*, Walkerton, Ontario. Emerg. Infect. Dis..

[19] Zhang X., Yin T., Du X., Yang W., Huang J., Jiao X. (2017). Occurrence and genotypes of *Campylobacter* species in broilers during the rearing period. Avian Pathol..

[20] Iglesias-Torrens Y., Miró E., Guirado P., Llovet T., Muñoz C., Cerdà-Cuéllar M., Madrid C., Balsalobre C., Navarro F. (2018). Population Structure, Antimicrobial Resistance, and Virulence-Associated Genes in *Campylobacter jejuni* Isolated from Three Ecological Niches: Gastroenteritis Patients, Broilers, and Wild Birds. Front. Microbiol..

[21] Wei B., Cha S.-Y., Kang M., Roh J.-H., Seo H.-S., Yoon R.-H., Jang H.-K. (2014). Antimicrobial susceptibility profiles and molecular typing of *Campylobacter jejuni* and *Campylobacter coli* isolates from ducks in South Korea. Appl. Environ. Microbiol..

[22] Wieczorek K., Osek J. (2017). Antimicrobial Resistance and Genotypes of *Campylobacter jejuni* from Pig and Cattle Carcasses Isolated in Poland During 2009–2016. Microb. Drug Resist..

[23] Lapierre L., Gatica M.A., Riquelme V., Vergara C., Yanez J.M., San Martin B., Saenz L., Vidal M., Martinez M.C., Araya P. (2016). Characterization of Antimicrobial Susceptibility and Its Association with Virulence Genes Related to Adherence, Invasion, and Cytotoxicity in Campylobacter jejuni and *Campylobacter coli* Isolates from Animals, Meat, and Humans. Microb. Drug Resist..

[24] Humphrey T., O’Brien S., Madsen M. (2007). Campylobacters as zoonotic pathogens: A food production perspective. Int. J. Food Microbiol..

[25] Tresse O., Alvarez-Ordonez A., Connerton I.F. (2017). Editorial: About the Foodborne Pathogen Campylobacter. Front. Microbiol..

[26] Koolman L., Whyte P., Burgess C., Bolton D. (2015). Distribution of virulence-associated genes in a selection of Campylobacter isolates. Foodborne Pathog Dis..

[27] Luangtongkum T., Jeon B., Han J., Plummer P., Logue C.M., Zhang Q. (2009). Antibiotic resistance in Campylobacter: Emergence, transmission and persistence. Future Microbiol..

[28] Paudyal N., Yue M. (2019). Antimicrobial Resistance in the “Dark Matter”. Clin. Infect. Dis..

[29] Engberg J., Aarestrup F.M., Taylor D.E., Gerner-Smidt P., Nachamkin I. (2001). Quinolone and macrolide resistance in *Campylobacter jejuni* and *C. coli*: Resistance mechanisms and trends in human isolates. Emerg. Infect. Dis..

[30] Li Y., Zhang S., He M., Zhang Y., Fu Y., Liang H., Jing H., Li Y., Ma H., Zhang M. (2018). Prevalence and Molecular Characterization *of Campylobacter spp*. Isolated from Patients with Diarrhea in Shunyi, Beijing. Front. Microbiol..

[31] Zhang M., Gu Y., He L., Ran L., Xia S., Han X., Li H., Zhou H., Cui Z., Zhang J. (2010). Molecular typing and antimicrobial susceptibility profiles of *Campylobacter jejuni* isolates from north China. Appl. Environ. Microbiol..

[32] Zhang A., Song L., Liang H., Gu Y., Zhang C., Liu X., Zhang J., Zhang M. (2016). Molecular subtyping and erythromycin resistance of *Campylobacter* in China. Appl. Environ. Microbiol..

[33] Li B., Ma L., Li Y., Jia H., Wei J., Shao D., Liu K., Shi Y., Qiu Y., Ma Z. (2017). Antimicrobial Resistance of *Campylobacter* Species Isolated from Broilers in Live Bird Markets in Shanghai, China. Foodborne Pathog. Dis..

[34] Zhang T., Luo Q., Chen Y., Li T., Wen G., Zhang R., Luo L., Lu Q., Ai D., Wang H. (2016). Molecular epidemiology, virulence determinants and antimicrobial resistance of *Campylobacter* spreading in retail chicken meat in Central China. Gut Pathog..

[35] Elbediwi M., Li Y., Paudyal N., Pan H., Li X., Xie S., Rajkovic A., Feng Y., Fang W., Rankin S.C. (2019). Global Burden of Colistin-Resistant Bacteria: Mobilized Colistin Resistance Genes Study (1980–2018). Microorganisms.

[36] Law J.W.-F., Ab Mutalib N.-S., Chan K.-G., Lee L.-H. (2015). Rapid methods for the detection of foodborne bacterial pathogens: Principles, applications, advantages and limitations. Front. Microbiol..

[37] National Health and Family Planning Commission of the People’s Republic of China (2007). Diagnostic Criteria for Infectious Diarrhea (WS 271–2007).

[38] Jiang Z., Paudyal N., Xu Y., Deng T., Li F., Pan H., Peng X., He Q., Yue M. (2019). Antibiotic Resistance Profiles of *Salmonella* Recovered from Finishing Pigs and Slaughter Facilities in Henan, China. Front. Microbiol..

[39] National Health and Family Planning Commission of the People’s Republic of China (2008). Diagnostic Criteria for Infectious Diarrhea (WS 287–2008).

[40] National Health and Family Planning Commission of the People’s Republic of China (2008). Diagnostic Criteria for Infectious Diarrhea (WS 289–2008).

[41] Nachamkin I., Nguyen P. (2017). Isolation of *Campylobacter* Species from Stool Samples by Use of a Filtration Method: Assessment from a United States-Based Population. J. Clin. Microbiol..

[42] Ribot E.M., Fitzgerald C., Kubota K., Swaminathan B., Barrett T.J. (2001). Rapid pulsed-field gel electrophoresis protocol for subtyping of *Campylobacter jejuni*. J. Clin. Microbiol..

[43] Centers for Disease Control (CDC) (2011). Standard Operating Procedure for PulseNet PGFE of Campylobacter Jejuni.

[44] Paudyal N., Pan H., Elbediwi M., Zhou X., Peng X., Li X., Fang W., Yue M. (2019). Characterization of *Salmonella* Dublin isolated from bovine and human hosts. BMC Microbiol..

[45] Elbediwi M., Pan H., Biswas S., Li Y., Yue M. (2020). Emerging colistin resistance in *Salmonella enterica* serovar Newport isolates from human infections. Emerg. Microbes Infect..

[46] Biswas S., Elbediwi M., Gu G., Yue M. (2020). Genomic Characterization of New Variant of Hydrogen Sulfide (H2S)-Producing *Escherichia coli* with Multidrug Resistance Properties Carrying the *mcr*-1 Gene in China †. Antibiotics.

[47] Bolger A.M., Lohse M., Usadel B. (2014). Trimmomatic: A flexible trimmer for Illumina sequence data. Bioinformatics.

[48] Bankevich A., Nurk S., Antipov D., Gurevich A.A., Dvorkin M., Kulikov A.S., Lesin V.M., Nikolenko S.I., Pham S., Prjibelski A.D. (2012). SPAdes: A new genome assembly algorithm and its applications to single-cell sequencing. J. Comput. Biol..

[49] Gurevich A., Saveliev V., Vyahhi N., Tesler G. (2013). QUAST: Quality assessment tool for genome assemblies. Bioinformatics.

[50] Seemann T. (2014). Prokka: Rapid prokaryotic genome annotation. Bioinformatics.

[51] Parker C.T., Quinones B., Miller W.G., Horn S.T., Mandrell R.E. (2006). Comparative genomic analysis of *Campylobacter jejuni* strains reveals diversity due to genomic elements similar to those present in C. jejuni strain RM1221. J. Clin. Microbiol..

[52] Page A.J., Taylor B., Delaney A.J., Soares J., Seemann T., Keane J.A., Harris S.R. (2016). SNP-sites: Rapid efficient extraction of SNPs from multi-FASTA alignments. Microb. Genom..

[53] Stamatakis A. (2014). RAxML version 8: A tool for phylogenetic analysis and post-analysis of large phylogenies. Bioinformatics.

[54] Letunic I., Bork P. (2016). Interactive tree of life (iTOL) v3: An online tool for the display and annotation of phylogenetic and other trees. Nucleic Acids Res..

