# Supplementary figures and images for: Epidemiological and Genomic Characterization of Campylobacter jejuni Isolates from a Foodborne Outbreak at Hangzhou, China
Source: Int J Mol Sci. 2020 Apr 24;21(8):3001. doi: 10.3390/ijms21083001 (PMC7215453; doi:10.3390/ijms21083001)

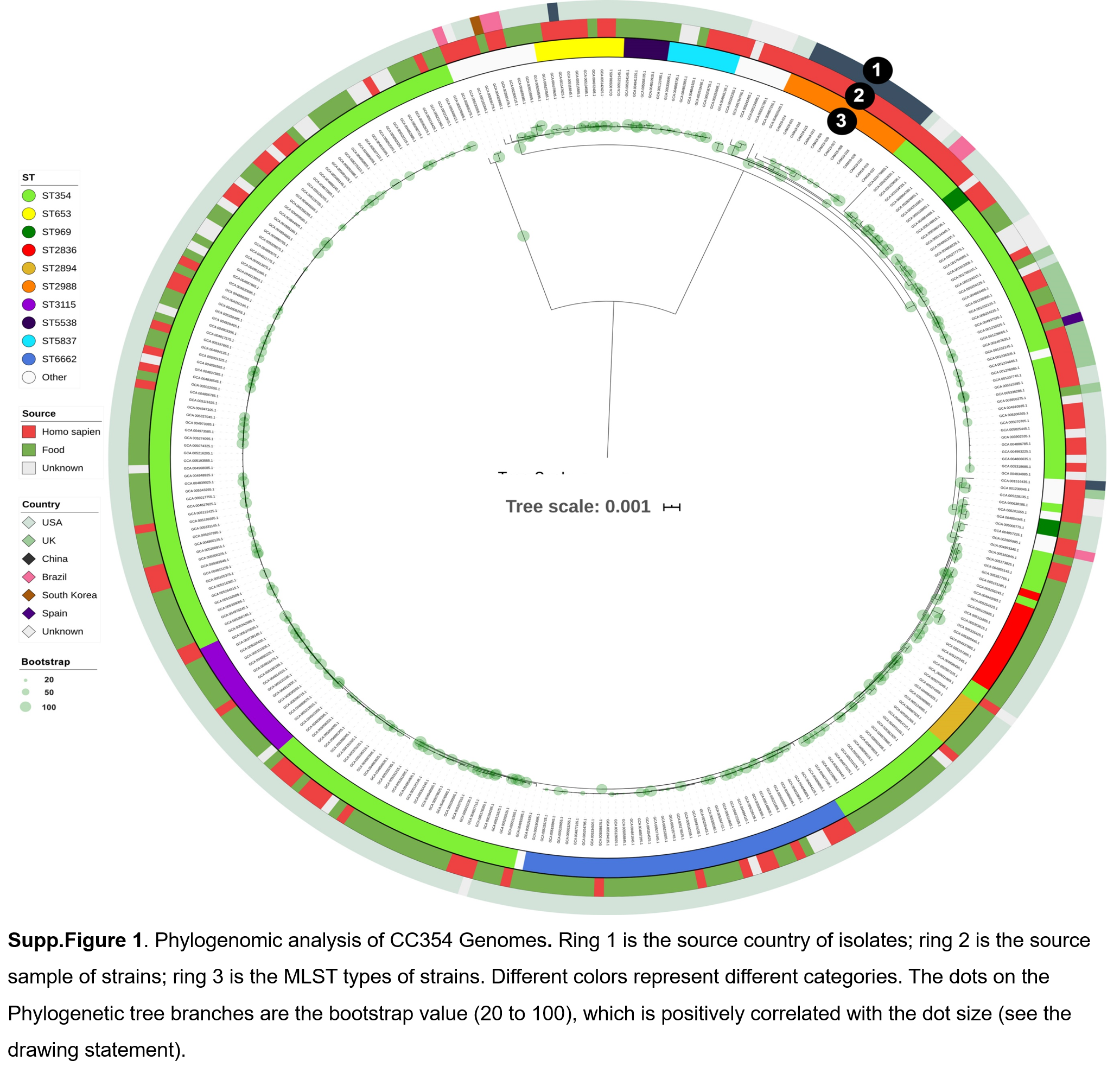

Supplement: Supplementary file 1 [file ijms-21-03001-s001.zip › Supplementary figure -1.tif]
